# One-year healthcare costs of patients with spontaneous intracerebral hemorrhage treated in the intensive care unit

**DOI:** 10.1177/23969873221094705

**Published:** 2022-04-29

**Authors:** Marika Smeds, Markus B Skrifvars, Matti Reinikainen, Stepani Bendel, Sanna Hoppu, Ruut Laitio, Tero Ala-Kokko, Sami Curtze, Gerli Sibolt, Nicolas Martinez-Majander, Rahul Raj

**Affiliations:** 1 Department of Emergency Care and Services, Helsinki University Hospital and University of Helsinki, Helsinki, Finland; 2 Department of Intensive Care, Kuopio University Hospital and University of Eastern Finland, Kuopio, Finland; 3 Department of Intensive Care, Tampere University Hospital and University of Tampere, Tampere, Finland; 4 Department of Department of Perioperative Services, Intensive Care and Pain Management, Turku University Hospital and University of Turku, Turku, Finland; 5 Department of Intensive Care, Oulu University Hospital and University of Oulu, Oulu, Finland; 6 Department of Neurology, Helsinki University Hospital and University of Helsinki, Helsinki, Finland; 7 Department of Neurosurgery, Helsinki University Hospital and University of Helsinki, Helsinki, Finland

**Keywords:** Stroke, cerebral hemorrhage, critical care, health care costs, cost of illness

## Abstract

**Background:**

Spontaneous intracerebral hemorrhage (ICH) entails significant mortality and morbidity. Severely ill ICH patients are treated in intensive care units (ICUs), but data on 1-year healthcare costs and patient care cost-effectiveness are lacking.

**Methods:**

Retrospective multi-center study of 959 adult patients treated for spontaneous ICH from 2003 to 2013. The primary outcomes were 12-month mortality or permanent disability, defined as being granted a permanent disability allowance or pension by the Social Insurance Institution by 2016. Total healthcare costs were hospital, rehabilitation, and social security costs within 12 months. A multivariable linear regression of log transformed cost data, adjusting for case mix, was used to assess independent factors associated with costs.

**Results:**

Twelve-month mortality was 45% and 51% of the survivors were disabled at the end of follow-up. The mean 12-month total cost was €49,754, of which rehabilitation, tertiary hospital and social security costs accounted for 45%, 39%, and 16%, respectively. The highest effective cost per independent survivor (ECPIS) was noted among patients aged >70 years with brainstem ICHs, low Glasgow Coma Scale (GCS) scores, larger hematoma volumes, intraventricular hemorrhages, and ICH scores of 3. In multivariable analysis, age, GCS score, and severity of illness were associated independently with 1-year healthcare costs.

**Conclusions:**

Costs associated with ICHs vary between patient groups, and the ECPIS appears highest among patients older than 70 years and those with brainstem ICHs and higher ICH scores. One-third of financial resources were used for patients with favorable outcomes. Further detailed cost-analysis studies for patients with an ICH are required.

## Introduction

Spontaneous (non-traumatic) intracerebral hemorrhage (ICH) accounts for approximately 10% to 15% of all strokes but has disproportionately high mortality.^1^ Despite attempts to find effective interventions, treatment options remain limited. Consequently, patient prognosis has not improved remarkably during the last decade.^2,3^ The one-year mortality rate is 40% to 50%, with a high proportion of survivors remaining severely disabled.^2,3^ The current AHA/ASA guidelines suggest initial treatment at dedicated neurological intensive care units (ICU) or stroke units, which places a significant economic burden on society, as care provided in these is expensive.^4^ Empirical evidence for intensive care’s cost-effectiveness in this patient group, however, remains unclear.

ICH incidence increases with age,^5^ which is also persistently implicated as a predictor of a poor prognosis.^6,7^ With an aging population, the number of patients affected and severely disabled by an ICH is likely to rise in the near future, leading to increasing treatment costs.^8–10^ Therefore, identification of those patients most likely to benefit from ICU care is needed to optimize allocation of health care funds. However, the epidemiological evidence on such patient characteristics remains scarce.^11^ In our previous study of the treatment cost-effectiveness of patients treated in the ICU following traumatic brain injury, subarachnoid hemorrhage, ischemic stroke, and hypoxic brain injury, spontaneous ICH was associated with the highest effective costs per independent survivor, costing approximately €180,000 altogether.^3^ This finding prompted a more detailed analysis of the factors driving 1-year healthcare costs and cost-effectiveness in patients with spontaneous ICH requiring ICU treatment.

## Methods

### Study design and population

**Figure 1.**
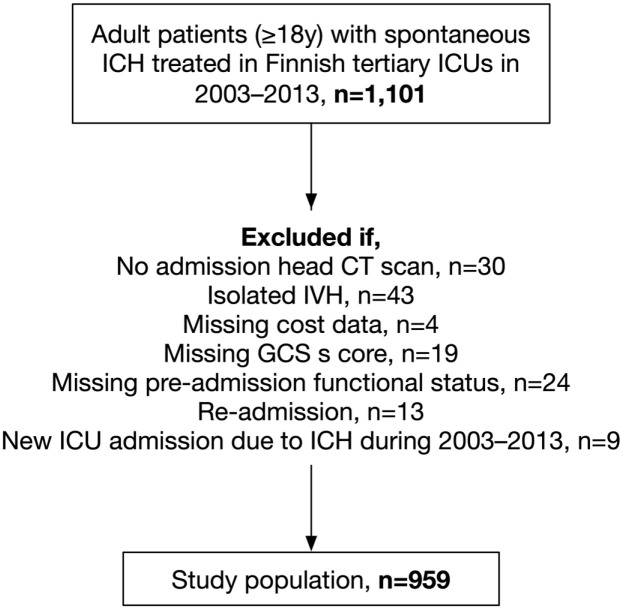
Study population. Abbreviations: CT indicates computed tomography; GCS, Glasgow Coma Scale score; ICH, intracerebral hemorrhage; ICU, intensive care unit; and IVH, intraventricular hemorrhage.

We adopted a retrospective multicenter design to study one-year healthcare costs and cost-effectiveness in patients with spontaneous ICH treated in ICUs. From the Finnish Intensive Care Consortium (FICC) database that has been collecting data prospectively since 1994,^12^ we extracted the records of adult patients (aged ⩾18) treated for spontaneous ICH in the five university hospital ICUs from January 1, 2003 through December 31, 2013. Patients with spontaneous ICH were identified based on their Acute Physiology and Chronic Health Evaluation III (APACHE II) diagnosis, and three of the authors, blinded to knowledge of the patient’s outcome, confirmed the diagnosis by manually reviewing the patients’ admission head computed tomography (CT) scans.^13^ If a patient had several entries in the FICC database due to ICH, we included the first admission. We excluded patients with isolated intraventricular hemorrhage (IVH) and incomplete data (Figure 1).

### Patient data

The data on demographic and clinical characteristics, treatment and intensive care scoring system scores (Simplified Acute Physiology Score II [SAPS II] and APACHE II)^14,15^ were extracted from the FICC database. Three authors (SC, N-MM, and GS) evaluated the hematoma volume (obtained by the ABC/2 method),^16^ the location, the extension to the ventricular system, and the midline shift from the admission head CT scan. The bleeding location was defined as supratentorial superficial, supratentorial deep, cerebellar, or brainstem. We also calculated the admission ICH score for each patient to stratify the risk of death.^6^ This score is based on radiological and clinical data, with points ranging from 0 to 6. Thirty-day mortality for those with the lowest score is predicted to be 0% and 100% for those with scores of 5 to 6.

### Outcome data

The primary outcome was death or permanent disability at 12 months. We obtained the date of death from the Finnish population register (available for all patients) up to the end of 2016. We used a surrogate marker for permanent disability if the patient was granted a permanent disability allowance or pension by the Social Insurance Institution in Finland by September 30, 2016. If the patient did not receive disability allowance, we assumed that the patient was independent in their daily activities. All Finnish citizens with a disability or long-term illness, regardless of personal insurance status, are entitled to a disability allowance from Kela. The criteria for granting a permanent disability allowance or pension are being unable to independently carry out activities of daily living or return to work due to sustained disability.^17^

### Cost data

We adjusted all costs for inflation, according to the Finnish Consumer Price Index (CPI), to 2021 euros, as follows ^18^:

$$\begin{array}{l} \mathrm{CPI}\;\mathrm{adjusted}\;\mathrm{cost}=\\ \mathrm{Cost}\;*\;\left(\mathrm{CPI}\;\mathrm{in}\;2021/\mathrm{Admission}\;\mathrm{year}\;\mathrm{CPI}\right) \end{array}$$

We examined total 12-month costs by assessing the university hospital, rehabilitation, and social security costs for 12 months after the admission. The hospital costs were obtained from the hospital billing departments and included expenses for ICU and ward stays, diagnostics (laboratory tests and radiological imaging), treatment received (including operations), and personnel. The rehabilitation costs were based on a report from the Finnish National Institute for Health and Welfare (THL) and determined according to the average cost of one ward day multiplied by the duration of the rehabilitation.^19^ The social security costs were extracted from the Kelas database and encompassed the costs of disability and sickness allowances, disability pensions, prescribed medication, travel expenses in connection to treatment, and expenses for private health care (physician and physiotherapist appointments). We assessed cost-effectiveness by dividing the total 12-month cost for all patients by the number of 12-month survivors and independent survivors to define the effective cost per survivor (ECPS) and per independent survivor (ECPIS).^20^

### Cost-effectiveness according to risk stratification

To identify patient characteristics associated with outcomes and costs, we repeated all outcome and cost analyzes separately for pre-defined outcome predictors, according to the ICH score and its components.^6^ As the prognoses of cerebellar and brainstem ICHs are vastly different we also considered bleeding locations in smaller subgroups (supratentorial superficial, supratentorial deep, cerebellar, and brainstem).^21^ In addition, we also report results according to age quintiles (five equally sized groups: 18–48, 49–57, 58–64, 65–70, and >70 years). Additionally, we report outcomes separately according to SAPS II score quartiles (four equally sized groups: 6–25, 26–39, 40–54, and 55–101).

### Statistical analysis

We used SPSS Statistics 27.0 for Mac OS (IBM Corp, Armonk, NY, USA) and Stata Statistical software for Mac OS (StataCorp LP, College Station, TX, USA) for all the statistical analyzes. We assessed differences in categorical subgroups with the Chi-squared (*χ*^2^) test. We tested the continuous data for the assumption of normality, and as the data were highly skewed, we employed the non-parametric Mann-Whitney *U* test or the independent *t*-test to compare subgroups as appropriate. The outcomes are presented as numbers and percentages for the categorical variables, means with 95% confidence intervals (CI) for the cost data, and medians with interquartile ranges (IQR) for all the other continuous variables. For the survival data, we also report 95% CIs. We considered *p*-values below 0.05 to indicate statistical significance.

Further, we assessed factors independently associated with increasing costs by linear regression, applying log transformation of the cost data to make the data conform to normality. We present the exponentiated regression coefficients as cost ratios.^22^ For example, a cost ratio of 1.1 indicates that the increase of one unit in the independent variable increase costs by 10%. The outcome predictors were defined a priori based on the ICH scores and our previous study on ICH long-term outcome predictors.^6,21^ We assessed collinearity by assessing the variance inflation factor (VIF). Separate models were built for the supratentorial and the infratentorial ICHs, and we correspondingly performed a subanalysis of hospital survivors only.

We also plotted the total costs against severity of illness. The severity of illness models were built in accordance with our previous study on ICH long-term outcome predictors,^23^ which for supratentorial ICH included the following: age, the Glasgow Coma Scale (GCS) score (defined as the lowest GCS score during the first 24 h after ICU admission or the last score preceding sedation for intubated/sedated patients, in accordance with the SAPS II criteria),^21^ the presence of a severe chronic comorbidity (based on the SAPS II and APACHE II definitions),^14,15^ the modified SAPS II score (SAPS II score points after excluding age, GCS, and chronic comorbidity points from the score),^14^ presence of IVH, the hematoma volume, and the midline shift. The infratentorial ICH severity of illness model was alike, except for including the hematoma location (cerebellar or brainstem) and excluding age, the midline shift, and hematoma volume.^21^ We subsequently applied the same technique to study hospital survivors only.

The methodological appraisal followed the Consolidated Health Economic Evaluation Reporting Standards (CHEERS) 2022 statement (Supplemental Material: Methods I).^24^

## Results

### Study population

Altogether, 959 patients met the study inclusion criteria (Figure 1). Most of the types of bleeding were supratentorial in origin (*n* = 786, 82%). The median patient age was 62 years, and most of the patients were independent in daily living prior to hospital admission (*n* = 873, 91%). Most of the patients underwent mechanical ventilation during their ICU stay (*n* = 524, 55%). The infratentorial ICH patients had smaller hematoma volumes but were more severely ill during the first day of hospital admission, according to their SAPS II scores, and were more likely to undergo mechanical ventilation or external ventricular drainage during their ICU stay. There were no major differences in age, severe comorbidities, treatment intensity, or length of ICU or hospital stay between the bleeding types. More detailed patient baseline characteristics are shown in Table 1.

**Table 1.** Patient baseline characteristics according to ICH location.

	All patients (*N* = 959)	Suprantentorial ICH (*n* = 786)	Infratentorial ICH (*n* = 173)	*p*-value*
Clinical variables				
Age, in years	62 (52–69)	60 (51–68)	63 (54–71)	0.069
18–48	183 (19)	156 (20)	27 (16)	0.220
49–57	188 (20)	154 (20)	34 (20)	
58–64	215 (22)	183 (23)	32 (18)	
65–70	170 (18)	134 (17)	36 (21)	
> 70	203 (21)	159 (20)	44 (25)	
⩾ 80	38 (4)	29 (4)	9 (5)	0.356
GCS score, median (IQR)	8 (4–13)	8 (4–13)	6 (3–13)	0.039
13–15	286 (30)	234 (30)	52 (30)	0.004
5–12	401 (42)	345 (44)	56 (32)	
3–4	272 (28)	207 (26)	65 (38)	
Severe chronic comorbidity†	109 (11)	93 (12)	16 (9)	0.332
Chronic anticoagulation	146 (15)	122 (16)	24 (14)	0.585
Pre-admission functional ability‡	8 (4–13)			
Independent	873 (91)	719 (91)	154 (89)	0.306
Dependent	86 (9)	67 (9)	19 (11)	
Admission year	2009 (2006–2011)	2009 (2006–2011)	2009 (2006–2011)	0.220
Radiological variables¶				
Location				
Supratentorial superficial	448 (47)	448 (57)	NA	NA
Supratentorial deep	338 (35)	338 (43)	NA	
Cerebellar	102 (11)	NA	102 (59)	
Brainstem	71 (7)	NA	71 (41)	
Hematoma volume, in ml	12 (5–28)	15 (6–34)	7 (3–12)	<0.001
Volume ⩾ 30 ml	223 (23)	219 (28)	4 (2)	<0.001
Midline shift, in mm	0 (0–7)	0 (0–8)	0 (0–0)	<0.001
⩾5 mm	317 (33)	314 (40)	3 (2)	<0.001
Intraventricular hemorrhage	425 (44)	351 (45)	74 (43)	0.652
ICH score§				
ICH score, median (IQR)	2 (1–3)	2 (1–3)	3 (2–3)	<0.001
0	144 (15)	144 (18)	0 (0)	<0.001
1	253 (26)	215 (27)	38 (22)	
2	250 (26)	209 (27)	41 (24)	
3	204 (21)	149 (19)	55 (32)	
4	101 (11)	67 (8)	34 (19)	
5	7 (1)	2 (1)	5 (3)	
6	0 (0)	0 (0)	0 (0)	
ICU variables				
Mechanical ventilation	524 (55)	420 (53)	104 (60)	0.007
Platelets, in E9/l	203 (157–251)	202 (157–248)	209 (158–261)	0.231
ICP monitoring	126 (13)	99 (13)	27 (16)	0.288
Modified SAPS II score**	16 (8–22)	15 (8–21)	17 (11–23)	0.031
SAPS II score	39 (25–54)	38 (25–53)	45 (27–57)	0.009
TISS-76 daily average score	26 (19–31)	26 (19–31)	27 (20–31)	0.738
External ventricular drain	118 (12)	75 (10)	43 (25)	<0.001
Duration of stay, in days				
ICU	2 (1–3)	2 (1–3)	2 (1–4)	0.639
Hospital	6 (3–12)	7 (3–12)	5 (2–13)	0.075

Categorical data presented as *n* (%), continuous data as median (IQR).

Abbreviations: GCS, Glasgow Coma Scale; ICH, intracerebral hemorrhage; ICP, intracranial pressure; ICU, intensive care unit; IQR, interquartile range; NA, not applicable; SAPS II, Simplified Acute Physiology Score II; TISS-76, Therapeutic Intervention Scoring System 76.

* Between supra and infratentorial ICH.

† Any chronic comorbidity according to Acute Physiology and Chronic Health Evaluation II and SAPS II score.

‡ A modified World Health Organization/Eastern Cooperative Oncology Group classification system implemented by the Finnish Intensive Care Consortium.

¶ Measured from admission head computerized tomography scan.

§ Based on GCS score (3–4 2p, 5–12 1p, 13–15 0p), age (⩾80 years 1p), ICH volume (⩾30 ml 1p), IVH (yes 1p) and ICH origin (infratentorial 1p, supratentorial 0p).

** SAPS II score without age, GCS score or chronic comorbidities.

The unadjusted outcomes for the different subgroups are shown in Table 2. The 12-month mortality was 45%. Of 531 patients surviving 12 months, 51% were left permanently disabled, and, hence, only 27% of all patients had a favorable outcome. Of the 428 deaths, 36% occurred during the ICU stay and 61% before hospital discharge. Both the short- and long-term mortalities were higher for the infratentorial ICH patients. Likewise, the functional outcomes differed significantly (*p* = 0.003) by ICH location: the proportion of patients alive and without permanently disability at 12 months was 17% for brainstem (*n* = 12), 22% for deep supratentorial (*n* = 74), 28% for cerebellar (*n* = 28), and 32% for superficial supratentorial (*n* = 144) ICHs.

**Table 2.** Unadjusted outcomes according to subgroups.

	30-day mortality	12-month mortality	Permanent disability in 12-month survivors
All patients	37% (33–40)	45% (42–48)	51% (47–56)
Supratentorial ICH	35% (31–38)	43% (40–47)	51% (46–56)
Superficial	30% (25–34)	39% (35–44)	47% (41–53)
Deep	42% (36–47)	49% (44–54)	57% (50–65)
Infratentorial ICH	45% (37–52)	50% (43–58)	54% (42.7–64)
Cerebellar	29% (20–38)	36% (27–46)	57% (45–69)
Brainstem	66% (55–78)	70% (60–81)	43% (20–66)
Age quintiles			
18–48	34% (28–41)	39% (32–46)	41% (32–50)
49–57	41% (34–48)	45% (38–52)	50% (40–59)
58–64	34% (28–40)	40% (33–46)	52% (44–61)
65–70	35% (28–42)	47% (40–55)	48% (37–58)
> 70	38% (32–45)	53% (46–60)	68% (58–77)
⩾ 80	29% (14–44)	42% (26–59)	73% (53–93)
GCS score			
13–15	5% (3–8)	13% (9–17)	42% (36–48)
5–12	32% (27–36)	42% (37–47)	59% (52–65)
3–4	77% (71–82)	82% (77–87)	65% (52–79)
ICH volume			
<30 ml	29% (26–32)	37% (34–42)	48% (44–53)
⩾30 ml	61% (55–67)	69% (63–75)	73% (62–84)
IVH			
No	26% (22–30)	33% (29–37)	51% (46–56)
Yes	50% (45–54)	59% (54–64)	52% (45–60)
ICH score*			
0	7% (3–11)	12% (7–17)	42% (33–50)
1	12% (8–15)	21% (16–26)	46% (39–53)
2	35% (29–41)	45% (39–51)	58% (50–66)
3	62% (56–69)	73% (66–79)	68% (55–81)
4	90% (84–96)	92% (87–97)	100% (100–100)
5	86% (51–121)	86% (51–121)	100% (100–100)
SAPS II quartiles†			
q1	4% (1–6)	8% (5–12)	40% (34–47)
q2	20% (14–25)	30% (24–36)	58% (51–66)
q3	48% (41–54)	60% (53–66)	61% (51–71)
q4	76% (71–81)	82% (77–87)	61% (45–76)

Outcome data presented as percentages with 95% confidence intervals (CI).

Abbreviations: GCS, Glasgow Coma Scale; ICH, intracerebral hemorrhage; IVH, intraventricular hemorrhage; SAPS II, Simplified Acute Physiology Score II.

* Based on GCS score (3–4 2p, 5–12 1p, 13–15 0p), age (⩾80 years 1p), ICH volume (⩾ 30 ml 1p), IVH (yes 1p) and ICH origin (infratentorial 1p, supratentorial 0p). †Quartile 1 SAPS II score 6–25 (*n* = 244, 25.4%), quartile 2 score 26–39 (*n* = 236, 24.6%), quartile 3 score 40–54 (*n* = 242, 25.2%), quartile 4 score 55–101 (*n* = 237, 24.7%).

### Unadjusted costs

**Figure 2.**
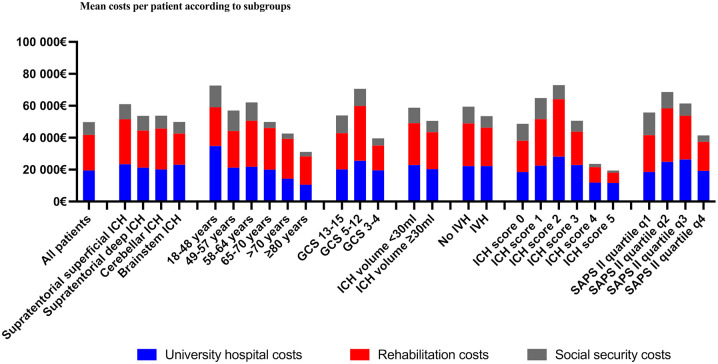
Mean costs per patient according to subgroups. Abbreviations: GCS indicates Glasgow Coma Scale score; ICH, intracerebral hemorrhage; IVH intraventricular hemorrhage; SAPS II, Simplified Acute Physiology Score II; q1, SAPS II score 6–25; q2, SAPS II score 26–39; q3, SAPS II score 40–54; and q4, SAPS II score 55–101.

The mean 12-month total cost per patient was €49,754, of which rehabilitation costs accounted for 45%, tertiary hospital costs for 39%, and social security costs for 16%. All costs were higher for the supratentorial ICH patients (total mean cost €50,654 for supratentorial ICH vs. 45,665€ for infratentorial ICH). The mean total costs were highest for the superficial supratentorial ICHs, followed by the cerebellar, deep supratentorial and brainstem ICHs (€53,419, €47,074, €46,990, and €43,642, respectively). The total costs decreased with age: the total cost was €63,607 for those aged 18–48 years compared to €37,275 for those aged >70 years. Furthermore, the mean total costs were lower for those with severe ICH compared to those with less severe ICH (€17,035 for ICH score 5 patients vs €42,669 for ICH score 0 patients). Of all the summed costs, 32% was spent on patients with a favorable outcome at 12 months (the sum of all costs was €47,714,359, and the sum cost for the patients with favorable outcomes at 12 months was €15,183,922, *n* = 258). Figure 2 and Supplementary Table 1 contain more details.

### Cost-effectiveness

**Figure 3.**
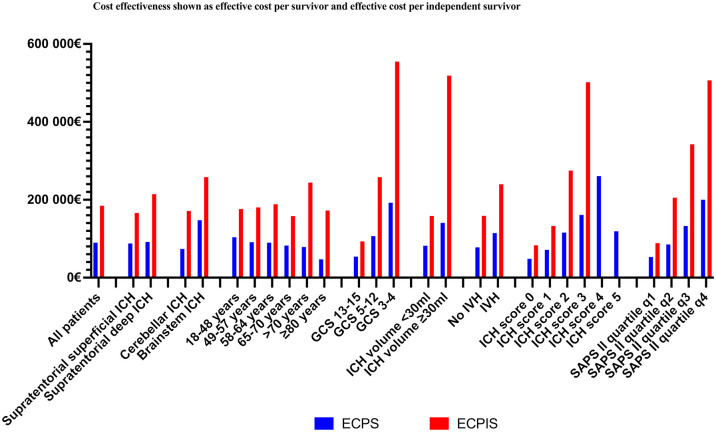
Effective cost per survivor (ECPS) and independent survivor (ECPIS) according to subgroups. Abbreviations: GCS indicates Glasgow Coma Scale score; ICH, intracerebral hemorrhage; IVH intraventricular hemorrhage; SAPS II, Simplified Acute Physiology Score II; q1, SAPS II score 6–25; q2, SAPS II score 26–39; q3, SAPS II score 40–54; and q4, SAPS II score 55–101.

The ECPS was €89,857 and the ECPIS was €184,940 for all patients. Both the ECPS and the ECPIS were higher for the infratentorial than the supratentorial ICHs: the ECPS was €91,861 and the ECPIS was €197,503 for infratentorial ICH compared to the ECPS of €89,470 and the ECPIS of €182,634 for the supratentorial ICH patients. The ECPS and the ECPIS were highest for the patients with brainstem ICH, a low GCS score, a larger ICH volume, and IVH. Although ECPIS was highest for those aged >70 years, the oldest age quintiles had the lowest ECPS. Figure 3 and Supplementary Table 2 contain more details.

### Factors associated with costs

In the multivariable analyzes (Table 3), deep ICH location, ICH volume ⩾30 ml, higher age and a lower GCS score were associated with lower costs, and a higher SAPS II score was associated with higher 12-month total costs among the supratentorial ICH patients (no notable collinearity, VIF_mean_ = 2.09). When considering supratentorial ICH hospital survivors only, higher age was associated with lower costs, whereas GCS 5–12, ICH volume ⩾30 ml and higher SAPS II score were associated with higher costs (no notable collinearity, VIF_mean_ = 1.78).

**Table 3.** Factors associated with healthcare costs based on multivariable ln-level linear regression analysis.

Group	All suprantentorial ICH (*n* = 786)				Suprantentorial ICH hospital survivors (*n* = 590)			
Variable	Cost ratio (e^B^)	95% CI		*p*-value	Cost ratio (e^B^)	95% CI		*p*-value
Location								
Superficial	Ref				Ref			
Deep	0.83	0.71	0.96	0.012	0.91	0.78	1.05	0.184
Age quintiles								
18–48	Ref				Ref			
49–57	0.86	0.69	1.08	0.198	0.84	0.67	1.05	0.123
58–64	0.87	0.70	1.08	0.217	0.78	0.63	0.97	0.024
65–70	0.70	0.55	0.89	0.003	0.56	0.45	0.71	<0.001
>70	0.56	0.44	0.70	<0.001	0.44	0.35	0.55	<0.001
GCS score								
13–15	Ref				Ref			
5–12	1.20	0.97	1.49	0.097	1.23	1.02	1.49	0.032
3–4	0.47	0.34	0.64	<0.001	0.98	0.71	1.35	0.892
ICH volume								
<30 ml	Ref				Ref			
⩾30 ml	0.78	0.66	0.92	0.003	0.90	0.75	1.07	0.226
IVH	0.95	0.82	1.11	0.551	1.16	1.00	1.35	0.045
SAPS II quartiles*								
q1	Ref				Ref			
q2	1.24	0.99	1.55	0.066	1.39	1.14	1.69	0.001
q3	1.14	0.86	1.49	0.363	1.58	1.23	2.03	<0.001
q4	1.00	0.73	1.38	0.992	1.53	1.11	2.12	0.010
Group	All infratentorial ICH (*n* = 173)				Infratentorial ICH hospital survivors (*n* = 108)			
Variable	Cost ratio (e^B^)	95% CI		*p*-value	Cost ratio (e^B^)	95% CI		*p*-value
Location								
Cerebellar	Ref				Ref			
Brainstem	1.27	0.88	1.84	0.204	0.97	0.65	1.44	0.877
Age quintiles								
18–48	Ref				Ref			
49–57	0.80	0.45	1.43	0.456	0.59	0.32	1.09	0.091
58–64	0.86	0.47	1.57	0.625	0.88	0.46	1.66	0.684
65–70	0.80	0.45	1.41	0.435	0.74	0.41	1.33	0.303
>70	0.75	0.43	1.32	0.322	0.49	0.27	0.87	0.015
GCS score								
13–15	Ref				Ref			
5–12	2.24	1.23	4.05	0.008	2.20	1.37	3.56	0.001
3–4	1.72	0.80	3.73	0.166	2.37	1.13	4.95	0.023
ICH volume								
<30 ml	Ref				Ref			
⩾30 ml	0.97	3.20	3.04	0.964	NaN	NaN	NaN	
IVH	0.83	0.58	1.19	0.317	0.82	0.57	1.18	0.282
SAPS II quartiles*								
q1	Ref				Ref			
q2	1.10	0.62	1.96	0.750	1.15	0.71	1.84	0.568
q3	0.42	0.20	0.89	0.024	0.97	0.50	1.88	0.937
q4	0.23	0.10	0.52	0.001	0.90	0.40	2.02	0.791

Abbreviations: CI, confidence interval; GCS, Glasgow Coma Scale; ICH, intracerebral hemorrhage; IVH, intracerebral hemorrhage; NA, not available; SAPS II, Simplified Acute Physiology Score II.

* Quartile 1 SAPS II score 6–25 (*n* = 244, 25.4%), quartile 2 score 26–39 (*n* = 236, 24.6%), quartile 3 score 40–54 (*n* = 242, 25.2%), quartile 4 score 55–101 (*n* = 237, 24.7%).

The significant independent contributors to costs differed between the supratentorial and the infratentorial ICHs. For the infratentorial ICHs, a lower GCS score was associated with higher costs, and a higher SAPS II score was associated with lower 12-month total costs (no notable collinearity, VIF_mean_ = 2.62). When considering infratentorial ICH hospital survivors only, a lower GCS score remained as a significant independent contributor to high costs. Further, age higher than 70 years as associated with lower costs (no notable collinearity, VIF_mean_ = 2.13).

**Figure 4.**
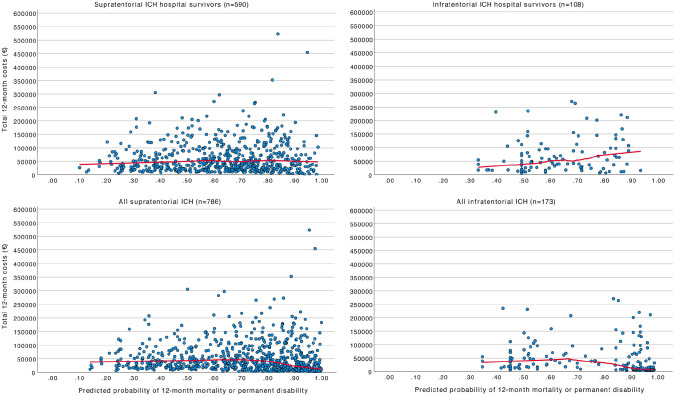
Scatterplot smoothing curves: Total 12-month costs according to illness severity for supratentorial and infratentorial ICH. Abbreviations: ICH indicates intracerebral hemorrhage.

Scatterplot smoothing (LOESS) curves in Figure 4 demonstrate the association between changes in total 12-month costs and illness severity. There was no clear positive correlation between total costs and illness severity; however, the most severe illness had a slight negative correlation with costs. However, considering hospital survivors only, the correlation with illness severity and costs was slightly positive. The same applied to the supratentorial and the infratentorial ICH patients.

## Discussion

This multicenter study demonstrated that spontaneous ICHs treated in the ICU were associated with substantial 1-year healthcare costs and, in many cases, poor outcomes. Most of the 1-year healthcare costs were associated with rehabilitation, and costs varied substantially between the different patient groups. The ECPIS was highest among the patients with older age, brainstem ICH, and more severe illness. The patient characteristics independently associated with costs also appeared to differ somewhat between the supratentorial and the infratentorial ICHs. It remains to be seen whether better triage and a focus on the appropriate patient groups could improve cost-effectiveness.

Previous studies have assessed the hospital-wide costs and mortality of spontaneous ICH, but data on a selected ICU cohort remain scarce. In a retrospective study on ICU-treated intracranial hemorrhage, Fernando et al. found the mean total costs for the hospital stay to be $53,491 (cost given in 2017 Canadian dollars, which converts to €37,164 at 2021 rates, including direct and indirect hospital costs) for ICH.^25^ However, ICH is also associated with a high rate of lifelong disability, which creates substantial economic expenses due to productivity losses.^5^ Furthermore, although recovery is more rapid in the first few weeks, it may continue for many months after ICH. Therefore, assessing outcomes and costs from a longer time perspective would provide a more comprehensive estimate of the cost-effectiveness and the total economic burden of ICH on society.^26^ In our population, 51% of the patients who survived for 12 months were left severely disabled, and only 27% of all the patients had a favorable outcome at 12 months. Our findings are in line with previous studies, as van Asch et al reported in a systematic review that the independence rate after ICH was 12–39%.^5^

We observed a mean total 12-month cost of €49,754, of which rehabilitation costs formed the biggest part. As we found the total costs to be substantially lower for those with a favorable outcome compared to those permanently disabled at 12 months (€58,852 vs €81,553), this emphasizes the importance and efficacy of investment in rehabilitation to improve and maintain functional ability, which is also in line with the current ICH guidelines.^4^ However, as only one-third of all costs was allocated to the patients with a favorable functional outcome, the resource allocation may not be optimal, and further studies on optimizing resource use are needed. Our previous study reported similar estimates with a mean total 12-month cost of €47,661 for ICH (costs shown in euros at the 2013 rate).^3^ However, compared to other critical illnesses, the mean total 12-month cost of ICH appears high.^17,27^

We found the lowest mean costs for the patients with a brainstem ICH, an ICH volume of >30 ml, an ICH score of 4–5, and a high SAPS II score (quantile 4), which all indicated severe ICH. As our previous study on ICH long-term outcome predictors showed, brainstem ICH was a strong predictor of mortality and was associated with a sharp drop in survival immediately after the insult.^21^ Furthermore, some previous studies have shown that approximately half of the deaths occur within the first two days.^23,28^ Due to the devastating prognosis of brainstem ICH, many patients require only a short ICU and hospital stay and receive fewer investigations and treatments before their inevitable death, leading to lower average total costs compared to other ICH subgroups. Interestingly, age over 70 was also associated with lower total costs, in line with the findings by Fernando et al.^25^ This could be explained in two ways. First, do not attempt resuscitation (DNAR) orders are significantly more common among older patients compared to younger age groups. Therefore, the oldest patients in the cohort likely had a favorable prognosis, as patients with poor prognoses may not have been referred to ICUs due to the presumed futility of care. Also, as previous studies have showed, early DNAR orders are a proxy of less active management and therefore might have self-fulfilling prophecies of poor outcomes and, accordingly, lower total long-term costs.^29,30^ In line with this hypothesis, we found the unadjusted index hospital costs and the ECPS to be lower among the older subgroups although mortality rates were similar to that of the younger age groups, indicating that the costs were lower because aggressive treatment was withheld. Further, elderly patients with ICH admitted to the ICU probably represent a very selected cohort of patients that have been admitted with a perceived high likelihood of recovery. Therefore, age-related costs should not determine the selection of patients for the ICU but should be considered when assessing total health care spending for ICH.

Among all the subgroups, we found the mean costs to be highest among those with a GCS of 5–12, no IVH, and an ICH score of 2, indicating that most resources are spent on subgroups with a moderate prognosis. Among these subgroups, recovery tends to require longer ICU and hospital stays, increased numbers of hospital interventions, and more intensive rehabilitation compared to those with a milder disease picture, hence leading to higher total costs.^31^ Furthermore, the mean costs were highest among the young (ages 18–48 years) among all bleeding locations, although for infratentorial ICH the association between costs and age was statistically significant when considering hospital survivors only. Together with a greater likelihood of an active treatment plan despite the severity of the illness discussed above, the expenses caused by a lifelong disability are higher among younger subgroups, which explains the age-related cost difference.

We also found that the ECPS and the ECPIS were highest for the patients with a GCS score 3–4, an ICH volume of >30 ml, an ICH score of 3, and a high SAPS II score (quantile 4). As these subgroups are associated with poor functional outcome despite high costs, it makes the cost-effectiveness of these subgroups questionable, which should be considered in resource allocation.

The costs associated with supratentorial and infratentorial ICHs differed somewhat. Low GCS scores were independently associated with lower costs among the supratentorial ICH patients, yet the opposite was true for infratentorial ICH. Similarly, high SAPS II scores were independently associated with higher costs among the survivors with supratentorial ICHs but with lower costs among all the infratentorial ICH patients. These findings reflect the differences in the disease picture and treatment options among the bleeding types. Overall, infratentorial bleeding is a well-known independent marker of poor prognosis, and as the very sick patients (i.e., with high SAPS II scores) die early, the costs remain low.^6^ Although a low GCS score associated with any location is a marker of poor prognosis, a patient with cerebellar ICH and impaired consciousness is more likely to undergo hematoma evacuation, which increases both survival and costs.^32^ In contrast, a low GCS score in patients with supratentorial ICH is often a sign of notable mass lesion and intracranial hypertension, which is seldomly treatable by for example surgical evacuation.

Like the study by Lekander et al., we found a minor correlation between ICH severity and costs when considering hospital and 12-month survivors only.^31^ This implies that functional ability is a determinant of the costs of ICH over 12 months, and, therefore, research should concentrate on treatments that could have a positive effect on functional outcome to reduce costs. However, those patients who are very sick die early and are therefore associated with lower costs.

### Strengths and limitations

Our study has several strengths. Given the multicenter study design that included all the tertiary hospitals in Finland and the tax-based Finnish healthcare system, the cohort is considered to capture population-based ICHs with no selection bias due to socioeconomic or insurance status. Additionally, after identifying cases by APACHE III diagnosis, all admission CT scans were screened to ensure the validity of the diagnoses and minimize misclassification bias. The databases used were high-quality, ensuring no loss to follow-up, and the data on cost status were almost 100% (99.6%).^12^ The cost data were gathered with a wide perspective from three resources covering rehabilitation and social security costs and costs of hospital care, offering a comprehensive estimate of the total economic cost of intensive care for ICH.

Some limitations also need to be acknowledged. First, due to the study’s retrospective nature, we were restricted to the data available in the databases. Therefore, we were unable to account for the effects of treatments, early treatment restrictions and withdrawal of care on costs. Hence, some patients in the study cohort might have been admitted to the ICU under consideration as potential organ donors. As early withdrawal of care might lead to decreasing total costs over a longer time perspective, withdrawal of treatment could be an important confounder in the association of illness severity and long-term costs. However, as we conducted a sensitivity analysis on hospital survivors only, the differences between supratentorial and infratentorial ICH remained. As the median duration of hospital care was 6 days and did not differ significantly among the supratentorial and the infratentorial ICH patients, the impact of withdrawal of care at an early phase is considered to have been covered in this subgroup analysis. Second, the modified Rankin Scale is the most common metric to estimate neurological outcome post-stroke.^33^ However, as a common intensive care database, FICC does not include neuro-specific data, and, hence, we used a surrogate marker for permanent disability for functional outcome, which was based on granted permanent disability allowances and pensions by the Social Insurance Institution. However, as all Finnish citizens with a disability or long-term illness are entitled to a disability allowance from Kela regardless of personal insurance status, we believe that the surrogate marker describes the functional outcome in our population well, although the surrogate marker carries a theoretical risk of both over- and underestimation of the patients’ functional status. A direct translation of our surrogate marker to the modified Rankin Scale is not possible, but it seems likely that a favorable outcome as defined by our surrogate outcome marker would translate into a mRS of 0–3 in most cases and into 0–4 in some instances. However, 1 year is a rather short time to assess functional outcome, and, in addition to the difference in reporting functional outcome, this should be considered when interpreting the results. Third, due to the data-derived restrictions, we used the ECPIS to measure cost-effectiveness instead of a quality-adjusted life year (QALY). However, the ECPIS is a rough estimation, and in addition to having a non-existent commonly described range, it cannot be directly translated to QALYs to make inferences. Nevertheless, there are similar studies in critical care in which cost-effectiveness has been described similarly, although there are no current guidelines on what can be considered as cost-effective ECPS or ECPIS values.^3,27^ More cost studies using similar metrics for other diseases are needed in order to establish such threshold values. For example, the ECPIS of ICU-treated ICH patients (€184,940) is lower than that of ICU-treated patients with acute ischemic stroke (€291,210^34^) but higher than that of ICU-treated patients with traumatic brain injury (€92,302^17^) and subarachnoid hemorrhage (€96,360^3^). However, ECPS and ECPIS do not account for indirect future costs, such as loss of ability to work. Fourth, most spontaneous ICH patients are treated at secondary and tertiary hospital stroke units in Finland instead of an ICU. As this study was based on patients treated at tertiary hospital ICUs only, the study cohort represents the patients comprising the more severe ICH cases that still were admitted to the ICU. For example, assuming an annual ICH incidence of 25 per 100,000 inhabitants, approximately 15,000 ICHs should have occurred during the 11-year study period in a population of 5.5 million inhabitants (ref).^5^ Thus, the included study population constitute approximately 6% of all ICH patients, which should be considered when interpreting these results. Fifth, the infratentorial ICH population was notably smaller than the supratentorial ICH population (786 vs 173), and, hence, the subgroup analyzes are potentially underpowered and prone to generating false-negative results. Sixth, it should also be acknowledged that the patients were treated during 2003–2013. There may have been changes in care even though guidelines on the management of ICH have remained largely unchanged.^4,35^ Finally, given the diversity in organizing healthcare, these findings might not be readily generalizable to countries with completely different healthcare systems.

## Conclusions

Costs associated with ICHs treated in ICUs varied substantially between different patient groups. The ECPIS was highest among the patients older than 70 years, brainstem ICH, and more severe illness. The patient characteristics independently associated with costs differed somewhat between supratentorial and infratentorial ICHs. With better triage and a focus on the appropriate patient groups, cost-effectiveness could increase. As only one-third of all costs were allocated to those with a favorable functional outcome, further detailed cost-analysis studies for patients with ICH are required.

## Supplemental Material

sj-docx-1-eso-10.1177_23969873221094705 – Supplemental material for One-year healthcare costs of patients with spontaneous intracerebral hemorrhage treated in the intensive care unit Click here for additional data file. Supplemental material, sj-docx-1-eso-10.1177_23969873221094705 for One-year healthcare costs of patients with spontaneous intracerebral hemorrhage treated in the intensive care unit by Marika Smeds, Markus B Skrifvars, Matti Reinikainen, Stepani Bendel, Sanna Hoppu, Ruut Laitio, Tero Ala-Kokko, Sami Curtze, Gerli Sibolt, Nicolas Martinez-Majander and Rahul Raj in European Stroke Journal
