# Supplementary material for: One-year healthcare costs of patients with spontaneous intracerebral hemorrhage treated in the intensive care unit
Source: Eur Stroke J. 2022 Apr 29;7(3):267–79. doi: 10.1177/23969873221094705 (PMC9446333; doi:10.1177/23969873221094705)
Supplement: sj-docx-1-eso-10.1177_23969873221094705 – Supplemental material for One-year healthcare costs of patients with spontaneous intracerebral hemorrhage treated in the intensive care unit [file sj-docx-1-eso-10.1177_23969873221094705.docx]

**Supplementary Table 1. Mean costs per patient according to subgroups**

|  | Total costs (€) | University hospital costs (€) | Rehabilitation costs (€) | Social security costs (€) |
| --- | --- | --- | --- | --- |
| All patients* | 49,754 (46,166-53,342) | 21,744 (19,899-23,589) | 24,966 (22,292-27,640) | 8,853 (7,941-9,765) |
| Supratentorial ICH | 50,654 (46,686-54,622) | 21,943 (19,911-23,974) | 25,486 (22,478-28,495) | 9,140 (8,124-10,156) |
| Superficial | 53,419 (47,873-58,965) | 22,856 (19,961-25,751) | 27,561 (23,179-31,943) | 9,239 (7,900-10,578) |
| Deep | 46,990 (41,404-52,575) | 20,732 (17,963-23,500) | 22,736 (18,830-26,642) | 9,009 (7,440-10,577) |
| Infratentorial ICH | 45,665 (37,208-54,123) | 20,843 (16,401-25,285) | 22,602 (16,827-28,377) | 7,552 (5,480-9,625) |
| Cerebellar | 47,074 (37,193-56,954) | 19,680 (14,763-24,597) | 25,027 (17,441-32,614) | 7,863 (5,418-10,308) |
| Brainstem | 43,642 (28,401-58,882) | 22,514 (14,167-30,862) | 19,117 (10,057-28,177) | 7,106 (3,403-10,808) |
| Age quintiles |  |  |  |  |
| 18–48 | 63,607 (53,148-74,065) | 34,007 (27,069-40,945) | 23,742 (18,195-29,289) | 13,285 (10,882-15,688) |
| 49–57 | 49,920 (41,879-57,960) | 20,697 (17,227-24,167) | 22,508 (16,461-28,555) | 12,544 (10,136-14,951) |
| 58–64 | 54,401 (46,210-62,592) | 21,311 (18,260-24,362) | 28,156 (21,229-35,083) | 11,286 (9,011-13,561) |
| 65–70 | 43,685 (36,801-50,568) | 19,492 (16,234-22,749) | 25,620 (19,639-31,602) | 3,673 (2,632-4,715) |
| > 70 | 37,275 (31,685-42,864) | 14,005 (11,578-16,432) | 24,419 (19,305-29,533) | 3,203 (2,282-4,123) |
| ≥ 80 | 27,220 (20,394-34,046) | 10,272 (8,287-12,258) | 17,362 (10,553-24,170) | 2,764 (1,987-3,541) |
| GCS score |  |  |  |  |
| 13–15 | 47,225 (42,093-52,357) | 19,680 (16,660-22,700) | 22,224 (18,636-25,811) | 10,836 (9,192-12,479) |
| 5–12 | 61,781 (56,079-67,483) | 24,997 (22,282-27,711) | 33,538 (28,979-38,096) | 10,460 (8,843-12,078) |
| 3–4 | 34,683 (27,268-42,098) | 19,120 (15,103-23,138) | 15,212 (10,012-20,413) | 4,400 (3,241-5,560) |
| ICH volume |  |  |  |  |
| < 30ml | 51,435 (47,375-55,495) | 22,334 (20,204-24,465) | 25,653 (22,667-28,639) | 9,453 (8,442-10,464) |
| ≥ 30ml | 44,208 (36,547-51,870) | 19,796 (16,104-23,488) | 22,699 (16,743-28,655) | 6,875 (4,817-8,933) |
| IVH |  |  |  |  |
| No | 52,067 (47,325-56,809) | 21,743 (19,230-24,257) | 26,128 (22,584-29,673) | 10,275 (8,957-11,592) |
| Yes | 46,849 (41,359-52,338) | 21,745 (19,022-24,469) | 23,506 (19,425-27,587) | 7,068 (5,860-8,275) |
| ICH score† |  |  |  |  |
| 0 | 42,669 (36,835-48,503) | 18,081 (14,718-21,443) | 19,216 (14,416-24,016) | 10,354 (8,210-12,499) |
| 1 | 56,769 (50,657-62,881) | 21,992 (19,038-24,947) | 28,494 (23,871-33,117) | 12,911 (10,888-14,934) |
| 2 | 63,864 (55,083-72,645) | 27,488 (22,560-32,415) | 35,210 (28,766-41,654) | 8,623 (6,766-10,480) |
| 3 | 44,288 (36,090-52,485) | 22,365 (18,363-26,366) | 20,430 (14,379-26,480) | 6,664 (4,736-8,593) |
| 4 | 20,670 (12,940-28,401) | 11,598 (8,359-14,837) | 9,422 (2,897-15,946) | 2,064 (1,307-2,822) |
| 5 | 17,035 (-1,332-35,403) | 11,347 (6,031-16,663) | 6,357 (-9,198-21,913) | 1,320 (-,031-2,671) |
| SAPS II quartiles‡ |  |  |  |  |
| q1 | 48,850 (43,535-54,166) | 18,153 (15,552-20,755) | 22,543 (18,696-26,389) | 13,858 (12,009-15,707) |
| q2 | 60,043 (53,243-66,844) | 24,321 (20,695-27,947) | 32,717 (27,344-38,090) | 10,016 (7,951-12,081) |
| q3 | 53,788 (46,029-61,546) | 25,759 (21,712-29,807) | 26,839 (21,390-32,287) | 7,470 (5,611-9,330) |
| q4 | 36,321 (27,984-44,657) | 18,776 (14,498-23,054) | 17,830 (11,422-24,238) | 3,955 (2,755-5,156) |
| Cost data presented as means with 95% confidence intervals (95%CI).  Abbreviations: GCS, Glasgow Coma Scale; ICH, intracerebral hemorrhage; IVH, intraventricular hemorrhage; SAPS II, Simplified Acute Physiology Score II  *Costs are adjusted to the consumer price index in Finland and shown in euros (€) at the 2021 rate. †Based on GCS score (3–4 2p, 5–12 1p, 13–15 0p), age (≥ 80 years 1p), ICH volume (≥ 30ml 1p), IVH (yes 1p) and ICH origin (infratentorial 1p, supratentorial 0p). ‡Quartile 1 SAPSII score 6–25 (n=244, 25.4%), quartile 2 score 26–39 (n=236, 24.6%), quartile 3 score 40–54 (n=242, 25.2%), quartile 4 score 55–101 (n=237, 24.7%). | | | | |

**Supplementary Table 2. Cost-effectiveness shown as effective cost per survivor and effective cost per**

**independent survivor**

|  | ECPS (€) | ECPIS (€) |
| --- | --- | --- |
| All patients* | 89,857 (86,274-93,441) | 184,940 (181,356-188,522) |
| Supratentorial ICH | 89,470 (85,509-93,432) | 182,634 (3,962-178,672) |
| Superficial | 87,984 (82,454-93,515) | 166,193 (160,662-171,723) |
| Deep | 91,807 (86,241-97,372) | 214,630 (209,064-220,195) |
| Infratentorial ICH | 91,861 (83,463-100,260) | 197,503 (189,104-205,900) |
| Cerebellar | 73,870 (64,108-83,632) | 171,484 (161,721-181,245) |
| Brainstem | 147,550 (132,573-162,528) | 258,213 (243,235-273,190) |
| Age quintiles |  |  |
| 18–48 | 103,929 (93,540-114,318) | 176,364 (165,976-186,753) |
| 49–57 | 91,115 (83,127-99,104) | 180,479 (172,490-188,467) |
| 58–64 | 89,971 (81,826-98,116) | 188,649 (180,504-196,794) |
| 65–70 | 82,515 (75,682-89,350) | 158,009 (151,175-164,843) |
| > 70 | 78,821 (73,265-84,377) | 244,089 (238,534-249,646) |
| ≥ 80 | 47,016 (40,413-53,620) | 172,393 (165,791-178,997) |
| GCS score |  |  |
| 13–15 | 54,026 (48,914-59,136) | 93,148 (88,037-98,258) |
| 5–12 | 106,785 (101,100-112,471) | 258,065 (252,381-263,750) |
| 3–4 | 192,526 (185,144-199,909) | 554,929 (547,547-562,311) |
| ICH volume |  |  |
| < 30ml | 82,116 (78,064-86,170) | 158,392 (154,340-162,446) |
| ≥ 30ml | 140,835 (133,215-148,456) | 518,867 (511,247-526,487) |
| IVH |  |  |
| No | 77,881 (73,150-82,613) | 158,878 (154,147-163,609) |
| Yes | 114,429 (108,955-119,904) | 239,887 (234,414-245,361) |
| ICH score† |  |  |
| 0 | 48,381 (42,596-54,165) | 83,031 (77,247-88,816) |
| 1 | 71,455 (65,372-77,537) | 132,986 (126,903-139,068) |
| 2 | 115,695 (106,957-124,433) | 275,275 (266,536-284,013) |
| 3 | 161,333 (153,185-169,482) | 501,926 (493,778-510,075) |
| 4 | 260,964 (253,327-268,600) | NA‡ |
| 5 | 119,248 (104,536-133,960) | NA‡ |
| SAPS II quartiles¶ |  |  |
| q1 | 53,212 (47,922-58,501) | 88,952 (83,662-94,241) |
| q2 | 85,363 (78,597-92,128) | 205,367 (198,601-212,131) |
| q3 | 132,823 (125,103-140,543) | 342,543 (334,823-350,263) |
| q4 | 200,185 (191,892-208,479) | 506,351 (498,058-514,646) |
| Abbreviations: GCS, Glasgow Coma Scale; ECPIS, Effective cost per independent survivor; ECPS, Effective cost per survivor; ICH, intracerebral hemorrhage; IVH, intraventricular hemorrhage; NA, not available; SAPS II, Simplified Acute Physiology Score II  *Costs are adjusted to the consumer price index in Finland and shown in euros (€) at the 2021 rate. †Based on GCS score (3–4 2p, 5–12 1p, 13–15 0p), age (≥ 80 years 1p), ICH volume (≥ 30ml 1p), IVH (yes 1p) and ICH origin (infratentorial 1p, supratentorial 0p). ‡No independent survivors. ¶Quartile 1 SAPSII score 6–25 (n=244, 25.4%), quartile 2 score 26–39 (n=236, 24.6%), quartile 3 score 40–54 (n=242, 25.2%), quartile 4 score 55–101 (n=237, 24.7%). | | |
